# The role of produce surface topography on foodborne virus inactivation on polydimethylsiloxane topomimetic artificial leaf surfaces and fresh leafy green surfaces

**DOI:** 10.3389/fpls.2025.1636467

**Published:** 2025-08-28

**Authors:** Ashlyn Lightbown, Erin DiCaprio

**Affiliations:** Department of Food Science and Technology, University of California, Davis, Davis, CA, United States

**Keywords:** Tulane virus, leafy greens, sodium hypochlorite, peracetic acid, replicasts, polydimethylsiloxane, surface topography

## Abstract

Fresh leafy greens are a commodity known for high susceptibility to human norovirus contamination under pre- and post-harvesting conditions. However, the role of the leafy green surface topography on the attachment and removal of this pathogen is poorly understood. Romaine lettuce and spinach leaves from multiple plant stages of growth presenting variable microscale surface topographies were evaluated through the recovery of a human norovirus surrogate, Tulane virus (TV), and the impact on sanitizer inactivation. A previously developed model system using polydimethylsiloxane (PDMS) topomimetic artificial leaf surfaces (“replicasts”) was also used to elucidate the impact of produce surface topography on virus adhesion and inactivation, and to validate the use of this tool for produce safety-related studies. Overall, the leaf age or axis (adaxial or abaxial surface inoculated) did not influence the recovery of TV from romaine lettuce and spinach control samples (fresh or replicast). However, when evaluating the efficacy of two sanitizers commonly used in post-harvest wash water for leafy greens (sodium hypochlorite and peracetic acid), spinach leaf age impacted efficacy. Younger spinach leaves (15-day) had significantly higher inactivation (lower recovery) of infectious TV when treated with sodium hypochlorite compared to older leaves (45-day). The leaf axis did not impact TV inactivation in spinach. There was no influence of leaf age or axis on the inactivation of TV in romaine lettuce. Through modulation of replicast surface hydrophobicity utilizing surfactant addition to TV inoculum, we were able to mimic plant leaf hydrophobicity. It was found that romaine replicasts demonstrated comparable virus recovery to fresh plant tissue with 0.05% Tween 20 addition to TV inoculum. These data demonstrate that variations in microscale topography of spinach based on leaf age can influence the efficacy of sanitizers against TV, however this influence was not observed for romaine lettuce of either age or axis. Additionally, the data generated utilizing PDMS replicasts indicates these can be useful tools for studying foodborne virus recovery and inactivation in leafy greens.

## 1 Introduction

Human norovirus (NoV) is the leading causative agent of fresh produce-related foodborne outbreaks, and is estimated to cause 40% of illnesses attributed to produce reported in the United States each year. Viruses, as obligate intracellular parasites, are unable to replicate in the agri-food supply chain. All known foodborne viruses lack a lipid envelope, so the protein-composed viral capsid imparts enhanced resistance of the non-enveloped foodborne viruses to environmental stress (heat, UV exposure, desiccation) and chemical sanitizers (alcohol-based, chlorine-based, quaternary ammonium compounds) as compared to enveloped viruses or vegetative bacterial cells (Koopmans and Duizer, 2004). Therefore, foodborne viruses may persist for extended periods in food or the production environment following a contamination event. In addition, previous work has demonstrated that washing produce (with or without sanitizer addition) has limited efficacy on the inactivation or removal of foodborne viruses from produce surfaces (Bosch et al., 2018).

Human norovirus causes the majority of foodborne outbreaks and is responsible for a high number of fresh produce outbreaks in particular (Heaton and Jones, 2008; Seymour and Appleton, 2001). The greatest risk of human NoV infection comes from foods that undergo limited processing, such as fresh produce, that are processed without a sufficient “kill” step to inactivate virus in contaminated products. These commodities are also susceptible to cross-contamination with human NoV from food handler contact (Robilotti et al., 2015). An active area of study, work with human NoV and its surrogates has aimed to determine the mechanisms that contribute to the challenging removal of this virus from produce surfaces.

Ideally, studies aimed to characterize the interaction between foodborne viruses and produce surfaces would be conducted using plant tissues. However, there are inherent limitations to using plant tissues for such experiments due to the variability from plant to plant. To mitigate this variation, previous work has shown that topomimetic artificial leaf surfaces, i.e. replicated casts (‘replicasts’) of real leaves made from polydimethylsiloxane (PDMS), can be used in place of fresh plant tissue in such studies (Sun et al., 2019). Confocal and scanning electron microscopy of fresh leaves and their PDMS replicasts revealed faithful, high-resolution replication of various leaf structures, including veins, epidermal pavement cells, trichomes, and stomata (Doan et al., 2020b). The PDMS replicasts have several features that make them highly suitable for experimentation. First, they are compatible with a wide range of microscopy techniques and various sterilization and decontamination practices that permit gnotobiotic conditions and the re-use of replicasts (Doan et al., 2020b). At least four identical PDMS replicasts can be crafted from a single negative mold without degradation of topographical quality (Doan et al., 2020a). Finally, utilizing PDMS replicasts standardizes surface chemical properties, allowing for independent study of surface topography from surface hydrophobicity effects (Sun et al., 2019).

Several factors have been studied to elucidate the interactions between foodborne viruses and fresh produce and their resistance to removal through common washing procedures. Proposed mechanisms include non-specific binding of the viral particle to surfaces through ionic interactions, specific receptor-mediated attachment of the virus to produce surface moieties, or purely physical interactions relative to the size of the viral particle and the surface complexity of produce. The viral protein capsid will have an overall net positive or negative charge depending on its isoelectric point and the pH of the surrounding medium. Previous work has shown that by modulating the viral capsid electrostatic characteristics, absorption and desorption to produce surfaces can be altered (Tian et al., 2011; Vega et al., 2005, 2008). The cellular receptor recognized for human NoV attachment is histo-blood group antigens (HBGAs). Previously, human NoV genogroup II genotype 4 (GII.4) virus-like particles (VLPs) were found to attach to H-type HBGA-like carbohydrate components of romaine lettuce cell walls and this attachment was mediated by the human NoV HBGA binding domain (Esseili et al., 2019; Gao et al., 2016). Studies have also shown that foodborne viruses can accumulate in and around plant surface microstructures such as stomata and epidermal cells junctures. Human NoV, for example, has been found to localize around the stomata of fresh romaine lettuce, allowing for internalization and further dissemination of virus throughout edible portions of the plant (DiCaprio et al., 2015; Wei et al., 2010; Esseili et al., 2018). However, the role of microscale complexity of a produce surface on adhesion and/or dispersion of viruses has not been directly compared. The use of PDMS replicasts to evaluate the role of plant surface microscale topography reduces influence of multiple variables (native microbiota, plant cuticle, other plant surface moieties) on viral aggregation, leaving surface topography as the primary variable for evaluation. The aim of this work was to understand the impact that physical factors of produce surfaces have on virus inactivation and removal by chemical sanitizer washing.

## 2 Materials and methods

### 2.1 Fabrication of PDMS replicasts

Romaine lettuce (Lettuce, Parris Island Organic, Ferry-Morse, Norton, MA, U.S.) and spinach (Spinach, Space Hybrid, W. Atlee Burpee Co., Warminster Township, PA, U.S.) were grown from seed in a growth chamber with temperature, relative humidity, and photoperiod control to minimize leaf-to-leaf variability and damage from environmental conditions. The emergence of two true leaves was defined as germination day 0. Six romaine and spinach plants were used to generate PDMS replicasts at days 15 and 45 post germination. At each sampling point, three to six leaves were randomly selected from each plant. Harvested plant tissues were aseptically transported to the laboratory within one hour. For each species, PDMS replicasts were generated for both the abaxial (top) and adaxial (bottom) surface at each timepoint (day 15 and day 45).

PDMS replicasts of abaxial and adaxial sides of 15- and 45-day old leaves were fabricated in a previously described two-step molding process (6). In brief, the SYLGARD™ 184 Silicone Elastomer Kit (Dow Chemical Company, Midland, MI, U.S.) was used to create negative molds of fresh leaves. After curing for 48 hours, the negative molds were separated from the fresh plant tissue and cleaned with a 1:100 solution of Triton™ X-100 (Sigma-Aldrich, St. Louis, MO, U.S.). These negative molds were treated with UV light for 60 minutes before submersion in a 1000:1 solution of toluene to octadecyltrichlorosilane (Sigma-Aldrich, St. Louis, MO, U.S.) for five minutes. Negative molds were rinsed with absolute ethanol for another two minutes before drying completely. PDMS was poured onto the prepared negative molds and allowed to set at 25°C for 48 hours. The positive cast was then separated from the negative mold. Flat PDMS replicasts (no surface topography) were generated using glass microscope slides and were included as controls in all experiments.

### 2.2 Microscopic validation of PDMS replicasts

PDMS replicasts of 15- and 45-day old abaxial and adaxial romaine and spinach surfaces were viewed using 4x, 10x, and 20x objectives (Olympus UPlanFLN) with an inverted optical microscope (IX71, Olympus, Center Valley, PA). Images were captured using an affixed ORCA-ER digital camera (Hamamatsu, Japan) and were analyzed using Metamorph imaging software (version 7.7.2.0, Universal Imaging Corporation). Fresh leaves harvested at the same intervals post-germination were observed using light microscopy with the same magnification. Images of fresh leaves were compared to those of PDMS replicasts representing the same leaf ages and axis (abaxial or adaxial) to ensure comparability in surface macro- and micro-scale topography. High-resolution replication of various leaf structures, including veins, epidermal pavement cells, trichomes, and stomata were visually observed in replicasts and compared to fresh plant samples to ensure fidelity.

### 2.3 Contact angle measurements of replicasts and fresh leaves

Two different surfactants were utilized to modulate the contact angle of a droplet of phosphate-buffered saline (PBS) on replicasts, TWEEN^®^ 20 (Sigma-Aldrich, St. Louis, MO, U.S.) and Silwet^®^ L-77 (PlantMedia, bioWORLD, Dublin, OH, U.S.). 0.05% (v/v) Tween 20 or 0.2% (v/v) Silwet L-77 were mixed with PBS prior to contact angle evaluation. PBS without the inclusion of surfactant was used to evaluate the initial contact angle of replicasts and flat PDMS, as well as evaluate the contact angle of fresh plant tissues. For this study, contact angle was defined as the angle formed where liquid (PBS with or without surfactant) met the solid leaf surface (fresh or replicast). To evaluate contact angle, four 10 µL drops of solution were placed randomly on the adaxial or abaxial surfaces of replicasts or leaves. Images of each droplet on the surface were captured using an optical tensiometer (Theta Optical Tensiometer, Attention, Biolin Scientific, Stockholm, Sweden). The images were analyzed (OneAttension version1.8, Biolin Scientific, Stockholm, Sweden) to measure the contact angle of each sessile drop. The average contact angle of the four droplets on each sample was calculated.

### 2.4 Virus propagation and quantification

To propagate Tulane virus (TV) (obtained from Dr. Jianrong Li, Ohio State University), confluent monolayers of the monkey kidney cell line MK2-LLC (ATCC^®^ no. CCL-7™, Manassas, VA, U.S.) were cultured. MK2-LLC cells were cultured in reduced serum Minimum Essential Medium (Opti-MEM™, Gibco™, Thermo Fisher Scientific, Waltham, MA, U.S.), supplemented with 2% Fetal Bovine Serum (FBS, GenClone™, Genesee Scientific, Morrisville, NC, U.S.) at 37°C under a 5% CO_2_ atmosphere. For growing TV stock, MK2-LLC cells were washed with Hank’s Balanced Salt Solution (HBSS, Gibco™, Thermo Fisher Scientific, Waltham, MA, U.S.) and subsequently infected with TV at an MOI of 0.1. After one hour incubation at 37°C, 15 mL of Opti-MEM with 2% FBS were added. The virus was harvested 48 hours post-inoculation, and subjected to three freeze-thaw cycles, followed by centrifugation at 3,000 rpm for 20 minutes at 4°C.

To quantify TV, a plaque assay was performed in LLC-MK2 cells. Briefly, cells were seeded into six-well plates (Corning Life Sciences, Wilkes-Barre, PA) at a density of 10^6^ cells per well. After 24 hours of incubation at 37°C, MK2-LLC cell monolayers were infected with 400 μl of a 10-fold dilution series of TV and the plates were incubated for one hour at 37°C with gentle agitation every 15 minutes. The cells were each overlaid with 2.5 mL of Dulbecco’s Modified Eagle Medium (DMEM, Gibco™, Thermo Fisher Scientific, Waltham, MA, U.S.) containing 1% agarose (UltraPure™ LMP Agarose, Invitrogen, Waltham, MA, U.S.), 2% FBS, and Penicillin-Streptomycin (Gibco™, Thermo Fisher Scientific, Waltham, MA, U.S.). After incubation at 37°C and 5% CO_2_ for two days, the plates were fixed with 10% formaldehyde. The plaques were visualized by staining with crystal violet (0.05% w/v) after removal of overlay. Viral titer was expressed as mean log_10_ plaque forming unit (PFU)/mL ± standard deviation of all replicates.

### 2.5 Sanitizer inactivation of TV on PDMS replicasts and romaine and spinach leaves

Sodium hypochlorite (SH) (5% available chlorine, Spectrum Chemical Mfg. Corp., New Brunswick, NJ, U.S.) was diluted in Milli-Q water to achieve treatment concentrations of 50 ppm and 200 ppm. The concentration of free chlorine was determined using the ColorQ^®^ High Range Chlorine kit (LaMotte Company, Chestertown, MD, U.S.). Peracetic acid (PAA) (3.5% w/w, RICCA Chemical Company, Arlington, TX, U.S.) was diluted in Milli-Q water to achieve treatment concentrations of 20 ppm and 80 ppm. The concentration of PAA was determined using the Hydrogen Peroxide & Peracetic Acid kit (LaMotte Company, Chestertown, MD, U.S.). Once concentration was verified, each concentration of sanitizer was transferred to a 15 mL sterile conical tube.

Half-inch diameter coupons of adaxial and abaxial surfaces of replicast and fresh leaves of the same age were cut using a cork borer. TV stock was prepared to achieve a titer of 1×10^7^ PFU/mL. Coupons of replicasts and fresh leaves were placed in a biosafety cabinet and spot inoculated with 50 µl of virus stock on either the adaxial or abaxial surface to achieve a titer of 2×10^5^ per coupon. The inoculated coupons were air-dried in the biosafety cabinet for one hour prior to sanitizer treatment.

To determine viral recovery, inoculated coupons were transferred to a 15 mL conical tube containing 1 mL PBS supplemented with 0.05% Tween 20 and virus titer was determined in the solution by viral plaque assay. For sanitizer treated samples after one hour incubation, inoculated coupons were transferred to 15 mL conical tubes containing sanitizer or Milli-Q water (two-step wash control). Coupons were agitated in sanitizer solution for 30 seconds before adding 10 µl of 10% sodium thiosulfate (Sigma-Aldrich, St. Louis, MO, U.S.) to each tube to neutralize residual sanitizer. Sanitizer solutions were retained for viral enumeration by plaque assay. Following sanitizer treatment and subsequent neutralization, coupons were transferred to a 15 mL conical tube containing PBS with 0.05% Tween 20. Coupons were agitated in rinse solution for 30 seconds and virus titers in wash solution were determined by plaque assay. For the control one-step wash, inoculated coupons were transferred directly to this PBS/Tween 20 solution without an intermediate mock sanitizer step. Each treatment included three replicates and virus titer is expressed as the mean PFU/mL ± standard deviation including all replicates.

### 2.6 Surfactant addition to replicast inoculum

Based on the measured contact angle, viral inoculum used on replicasts of romaine leaves was supplemented with 0.05% Tween 20 to reduce surface hydrophobicity to comparable levels to that of fresh romaine leaves. No surfactant was added to the viral inoculum used on replicasts of spinach leaves. In further experiments, viral inoculum was supplemented with 0.2% Silwet L-77 for romaine replicasts instead of Tween 20 to intentionally reduce surface hydrophobicity to a greater degree.

### 2.7 Plaque assay of sanitizer and rinse solutions

Plaque assays on the collected sanitizer and rinse solutions were performed to evaluate the efficacy of sanitizers on viral inactivation. To prepare plates for plaque assay, two mL of confluent MK2 cells were added to each well of a six-well tissue culture plate and incubated for 24 hours. Serial dilutions were performed on both sanitizer and rinse solutions. Excess media was removed from wells of plates and 400 μl of appropriate serial dilution was added to each corresponding well. Plates were allowed to incubate at 37°C for one hour before addition of agarose overlay solution. Plates were then allowed to solidify before incubation at 37°C for 48 hours. Plates were then fixed with 10% formaldehyde solution for two hours and then stained with 1% crystal violet in 15% ethanol solution overnight. Plates were rinsed and plaques were counted to enumerate recovered infectious TV titer in plaque-forming units (PFU/mL).

### 2.8 Statistical analysis

Each contact angle measurement was repeated with a minimum of four replicate drops. Statistical analysis was performed using Microsoft Excel using the *t*-test, paired two sample for means, to evaluate differences in contact angle for replicasts and fresh leaves. All sanitizer experiments were performed in triplicate for both replicast and fresh leaf samples. Statistical analysis was performed using RStudio with R version 4.3.2 (The R Foundation for Statistical Computing, Vienna, Austria) to calculate one-way analysis of variance (ANOVA) and estimated marginal means (EMMs) between different sanitizers, ages, axes, and types. A *p*-value <0.05 was considered to be significant, and *p*-values were adjusted *post-hoc* using the Tukey HSD method.

## 3 Results

### 3.1 Contact angle measurements

It was found that flat PDMS replicasts without any surface topography had an average contact angle of 107.5° (**Table 1**). Fresh romaine lettuce leaves had an average contact angle of 73.7° to 82.1°, while fresh spinach leaves had an average contact angle ranging from 111.2° to 122.7°, depending on leaf age and axis. PDMS replicasts of romaine leaves were found to have contact angles from 76.6° to 99.2°, and spinach replicasts ranged from 93.2° to 113.4°. As the mean fresh romaine contact angle was below 90° hydrophobicity, a surfactant was added to the sessile drops used to measure contact angle of replicasts. With the addition of 0.05% Tween 20, the contact angle of flat replicasts was reduced to 74.4° and the range for romaine replicasts was 62.2° to 82.7°, allowing all replicasts regardless of age and axis to fall below 90° hydrophobicity. As the fresh spinach leaves had measured larger contact angles above 90°, spinach PDMS replicasts were not modified with a surfactant to reduce contact angle. To measure the impact of an organosilicone surfactant on leaf hydrophobicity, sessile drops used to measure contact angle were supplemented with 0.2% Silwet L-77. This reduced the contact angle of flat replicasts by over 80°, well below the average contact angle of fresh plant tissue. As a result of these findings, TV stock was supplemented with 0.05% Tween 20 before inoculation of all romaine PDMS replicasts. No surfactant was added to the TV stock used to inoculate spinach PDMS replicasts.

**Table 1:** Contact angle measurements for fresh and replicast leaves of spinach and romaine in degrees (°).

Surface Type				
Age and Axis	Fresh	PDMS	PDMS/Tween	PDMS/Silwet
Rl5T	78.0 ± 15.5	99.2 ± 16.2	82.7 ± 11.5	
Rl5B	74.7 ± 12.7	91.4 ± 5.2	78.2 ± 8.7	
R45T	73.7 ± 12.4	92.0 ± 12.3	62.2 ± 8	
R45B	82.1± 7.4	76.6 ± 3.4	62.6 ± 3.8	
S15T	116.6 ± 2.2	96.5 ± 7.5	70.4 ± 3.1	
Sl5B	114.8 ± 5.9	93.2 ± 9.6	71.9 ± 3.5	
S45T	122.7 ± 5.4	113.4 ± 6.3	72.7 ± 12	
S45B	111.2 ± 6.3	106.7 ± 1.9	71.0 ± 4.1	
Flat		107.5 ± 2.7	74.4 ± 2.2	24.4 ± 2.2

“R” indicates romaine, “S” indicates spinach, “15” indicates leaves collected 15 days post-germination, “45” indicates leaves collected 45 days post-germination, “T” indicates adaxial, and “B” indicates abaxial, “Flat” indicates control replicast without surface topography. PDMS/Tween indicates a 0.05% Tween 20 supplement to sessile drops used for contact angle analysis. PDMS/Silwet indicates a 0.2% Silwet L-77 supplement to sessile drops used for contact angle analysis. Standard deviation values for all replicates represented after “±”. No data was collected for leafy green replicasts PDMS/Silwet due to contact angles below measurable capacity of the machine.

### 3.2 Impact of surface topography on virus recovery and inactivation

**Figure 1:**
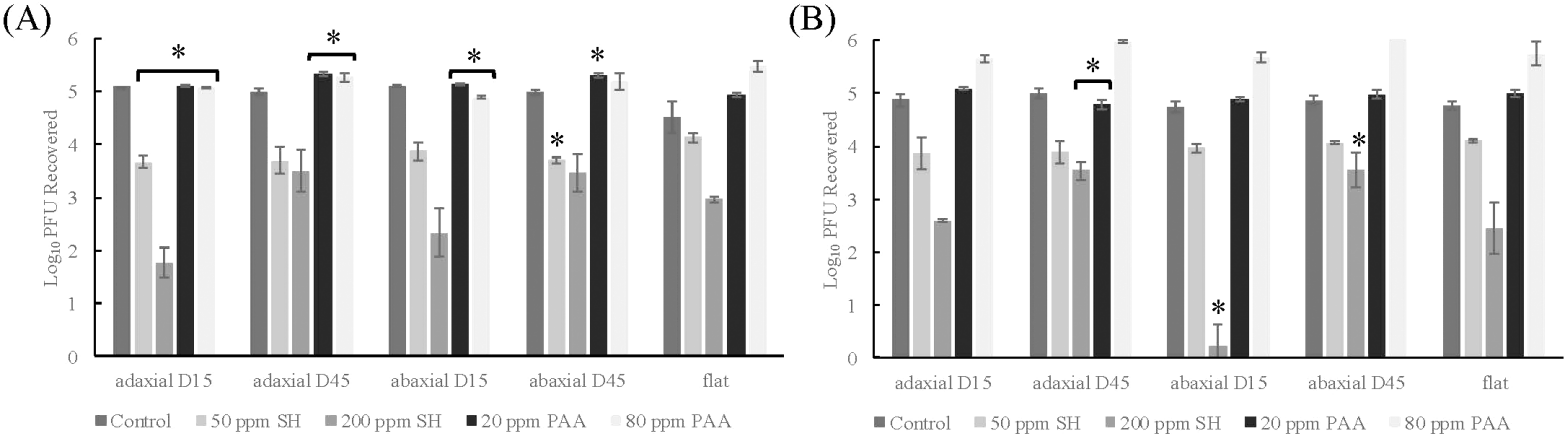
TV recovery from spinach, romaine replicasts, and flat replicasts after two-step washing procedure. **(A)** Spinach, **(B)** Romaine. Log_10_ PFU recovered is log-transformed Tulane virus titer recovered from coupons after a two-step washing procedure. Analysis of variance (ANOVA) with Tukey HSD method *post-hoc* applied to data to determine significance. *Denotes a significant difference (*p*-value <0.05) compared to respective treatment of flat replicasts.

PDMS coupons with no surface topography (flat) controls had a level of viral recovery of 4.5- 4.76 log_10_ PFU/coupon in the two-step washing procedure (mock sanitizer). In the spinach control, no significant differences were found in virus recovered between flat replicasts or any age or axis. In 15-day adaxial spinach replicasts, significant differences were observed between flat replicasts and all sanitizer treatments tested. In 15-day abaxial and 45-day adaxial replicasts, virus recovery for both PAA treatments was significantly lower when compared to flat replicasts (**Figure 1A**). In 45-day abaxial replicasts and flat casts, 50 ppm SH and 20 ppm PAA had significant differences in recovery compared to flat casts (*p*-values 0.013 and 0.018, respectively). All types of spinach replicasts displayed significantly lower virus recovery compared to flat replicasts.

In the romaine control, no significant differences were found in virus recovered between flat replicasts and romaine replicasts with no sanitizer addition. In 15-day adaxial romaine replicasts, no significant differences in virus recovery were observed between flat replicasts and any sanitizer treatment (**Figure 1B**). In 15-day abaxial and 45-day abaxial replicasts, treatment using 200 ppm SH led to significantly lower rates of viral recovery (*p*-values of 0.031 and 0.0074). In 45-day adaxial replicasts, 200 ppm SH and 20 ppm PAA treatments resulted in significantly lower viral recovery when compared to that from flat casts (*p*-values 0.046 and 0.018).

### 3.3 Virus recovery from replicasts and produce leaves

**Figure 2:**
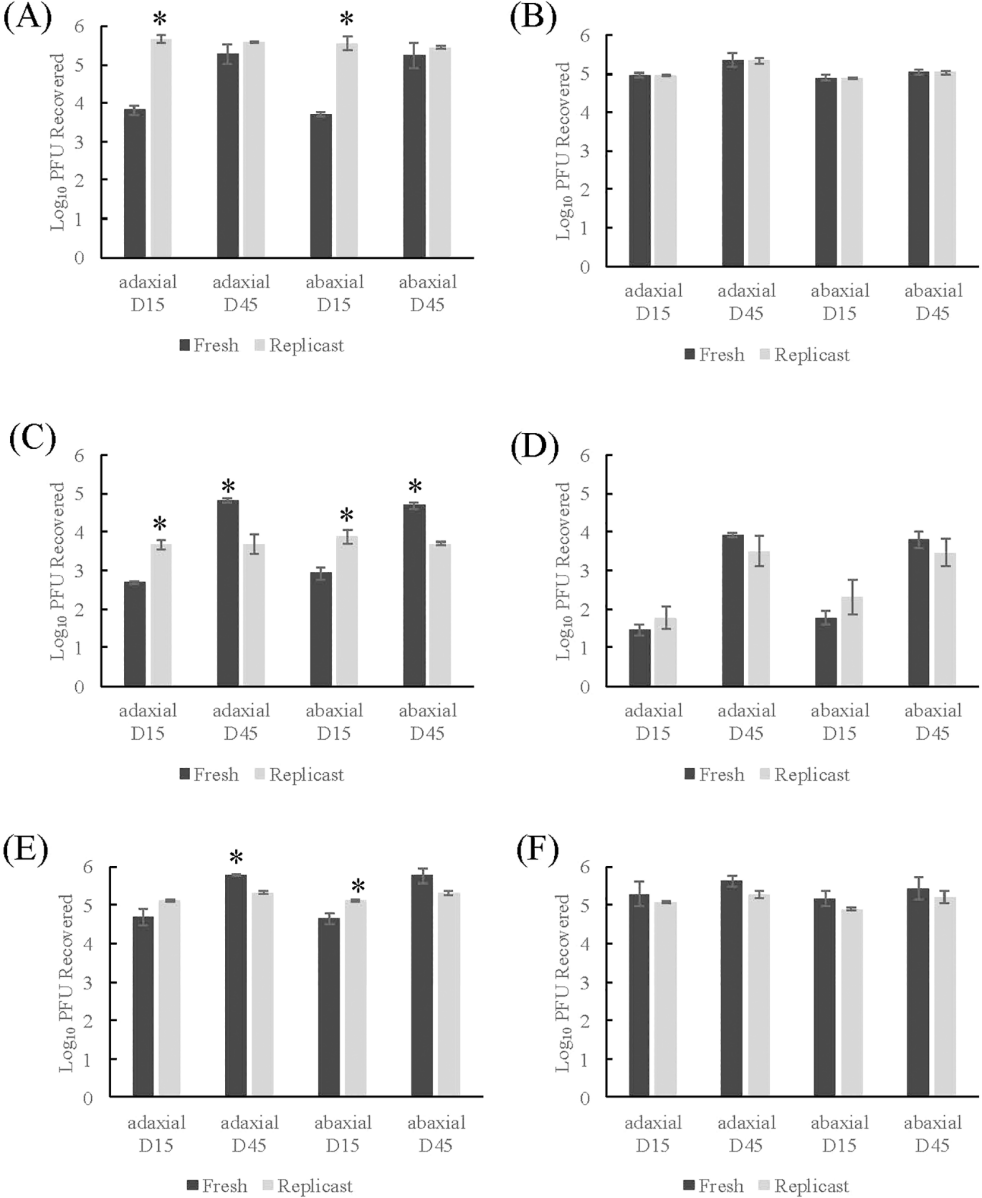
Comparison of TV recovery from spinach replicasts to fresh spinach after two-step washing procedure. **(A)** Control- one-step washing procedure, **(B)** Control- two-step washing procedure, **(C)** Treatment with 50 ppm sodium hypochlorite, **(D)** Treatment with 200 ppm sodium hypochlorite, **(E)** Treatment with 20 ppm PAA, **(F)** Treatment with 80 ppm PAA. Log_10_ PFU recovered is log-transformed Tulane virus titer recovered from coupons after a two-step washing procedure. Analysis of variance (ANOVA) with Tukey HSD method *post-hoc* applied to data to determine significance. *Denotes a significantly higher viral recovery (*p*-value <0.05) comparing fresh and replicast samples of same leaf age and axis.

Control TV-inoculated fresh spinach leaf coupons directly transferred to the PBS+0.05% Tween 20 wash solutions (one-step wash) had a level of virus recovery when inoculum was applied to adaxial spinach surfaces of 3.82 log PFU/mL (15-day) and 5.29 log PFU/mL (45-day) (**Figure 2A**). The replicasts of 15-day and 45-day spinach replicasts of the same leaf axis had viral titers of 5.68 and 5.59 log PFU/mL recovered, respectively (**Figure 2B**). The recovery of TV when applied to abaxial surfaces of 15- or 45-day spinach leaves and replicasts was not significantly different from the recovery from adaxial surfaces (**Figure 2B**). However, the recovery of TV from 15-day old spinach leaves was significantly lower (3.82 log PFU/mL adaxial; 3.71 PFU/mL abaxial) than the recovery of the corresponding replicast (5.68 log PFU/mL adaxial; 5.56 log PFU/mL abaxial) (*p*-value of 0.003 for both axes) (**Figure 2A**). For the mock sanitizer control samples, where TV inoculated coupons were transferred to Milli-Q water prior the wash solution, the recovery of TV was close to the inoculum level applied (2 ×10^5^ PFU/coupon) and the level was consistent across leaf age and axis for both spinach leaves and replicasts (**Figure 2B**).

**Figure 3:**
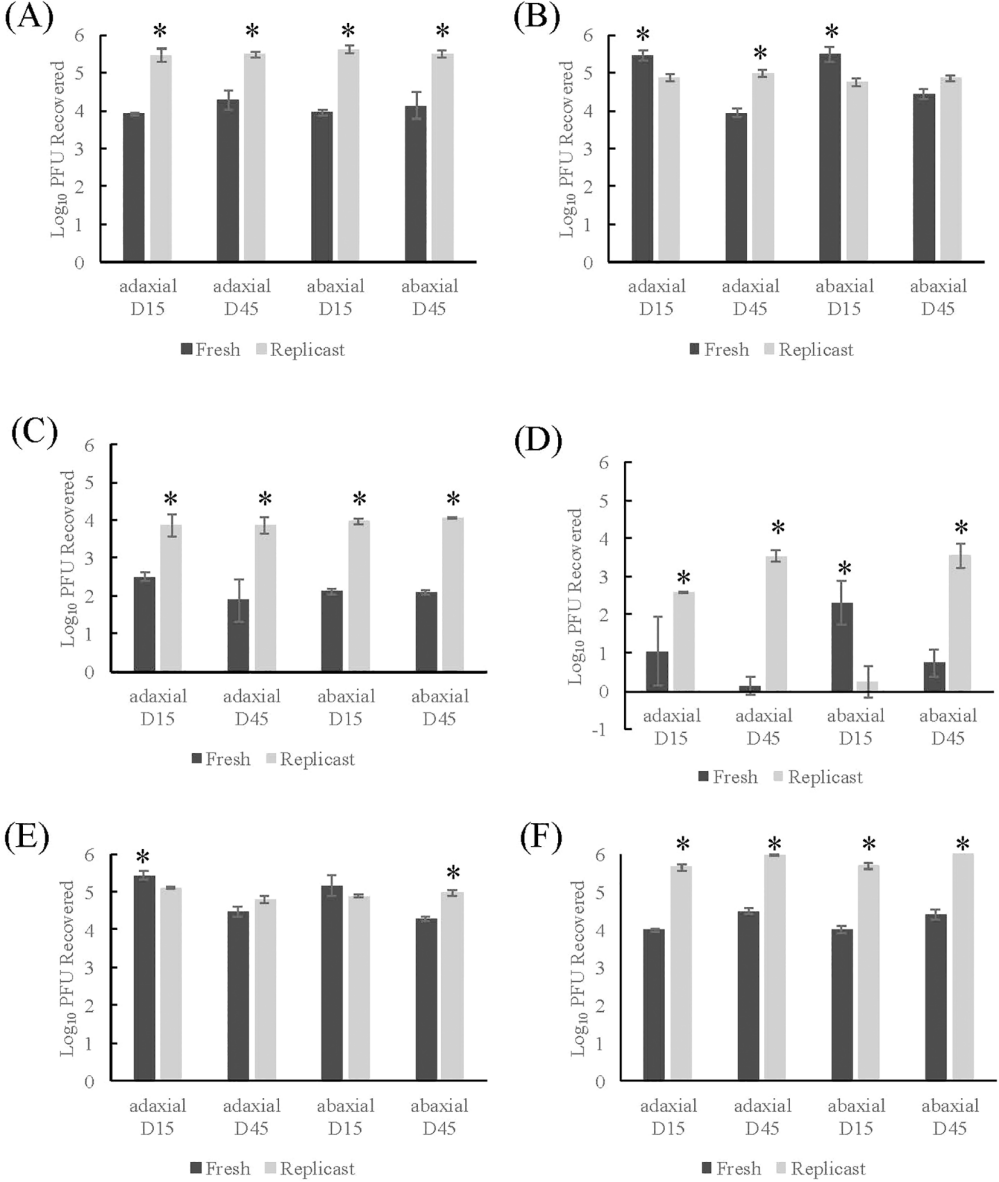
Comparison of TV recovery from romaine replicasts to fresh romaine after two-step washing procedure. **(A)** Control- one-step washing procedure, **(B)** Control- two-step washing procedure, **(C)** Treatment with 50 ppm sodium hypochlorite, **(D)** Treatment with 200 ppm sodium hypochlorite, **(E)** Treatment with 20 ppm PAA, **(F)** Treatment with 80 ppm PAA. Log_10_ PFU recovered is log-transformed Tulane virus titer recovered from coupons after a two-step washing procedure. Analysis of variance (ANOVA) with Tukey HSD method *post-hoc* applied to data to determine significance. *Denotes a significantly higher viral recovery (*p*-value <0.05) comparing fresh and replicast samples of same leaf age and axis.

Control fresh romaine lettuce leaf coupons transferred to the PBS+0.05% Tween 20 wash solutions (one-step wash) had a range of recovery from 3.91 to 4.29 log PFU/mL for 15- and 45-day old leaves of both axes (**Figure 3A**). The control replicasts had recovery close to the inoculum (2×10^5^ PFU/coupon) across all ages and axes and the recovery from replicasts was significantly higher than leaf samples (*p*-value <0.02). In mock sanitizer control samples (two-step wash), TV recovery from romaine leaves inoculated on adaxial surfaces was significantly different for 15-day (5.47 log PFU/mL) and 45-day old (3.94 log PFU/mL) leaves (**Figure 3B**). There was no statistical difference in the recovery of TV from romaine lettuce replicasts between age and axis or between 45-day old abaxial samples. There were, however, significant differences between replicasts and fresh leaves in 15-day old leaves or 45-day old adaxial leaves (*p*-value <0.05) with greater recovery in fresh than replicast for 15-day old leaves and greater recovery in replicast than fresh for 45-day old leaves. Overall, for both fresh and replicast spinach and romaine lettuce, viral recovery was enhanced using the mock sanitizer process compared to a single washing step.

### 3.4 Efficacy of sodium hypochlorite and peracetic acid on inactivation of TV on leaves and replicasts of varying surface topographies

The mock sanitizer control samples were used as the baseline to determine viral inactivation. In both spinach leaves and replicasts representing all ages and axes, there was no significant reduction in viral recovery based on treatment with either 20 ppm or 80 ppm PAA (**Figures 2E, F**). Treatment with 50 ppm SH led to a 1.97-2.28 log reduction in 15-day fresh leaves and a 1.23-1.42 log reduction in replicasts compared to the control (**Figure 2C**). There was no significant difference based on leaf axis. In 45-day leaves and replicasts, the 50 ppm SH treatment led to a 0.36-0.52 log and 1.30-1.31 log reduction, respectively. Again, there was no difference based on leaf axis. In both leaves and replicasts, there was a significant difference in the level of TV recovered from 15-day old samples compared to 45-day (*p*-value < 0.0001).

Higher SH treatment (200 ppm) resulted in a 3.13-3.52 and 2.78-3.33 log reduction in 15-day spinach leaves and replicasts, respectively. In 45-day old spinach leaves and replicasts, the level of reduction was 1.25-1.41 and 1.50-1.54 logs, respectively. For both ages of leaves and replicasts, there was no significant difference in viral recovery based on leaf axis. Similar to the low concentration of SH, the level of TV recovery was higher in older (45-day) leaves and replicasts compared to 15-day samples (**Figure 2D**) (*p*-value < 0.0001).

Similar to spinach samples, little to no inactivation of TV in fresh or replicast romaine lettuce was seen with either concentration of PAA regardless of leaf age or axis (**Figures 3E, F**). However, with a higher concentration of PAA treatment (80 ppm), significantly less infectious TV was recovered from fresh samples than replicast. Treatment of 15-day old leaves and replicasts with 50 ppm SH led to a 2.98-3.38 and 0.78-1.01 log reduction, respectively (**Figure 3C**). In 45-day old leaves and replicasts, the level of reduction was 2.06-2.34 and 0.8-1.11 logs, respectively. Similarly to results from the high concentration of PAA, less virus was recovered from fresh romaine than replicast. No significant difference in viral recovery was observed based on leaf age or axis in fresh or replicast romaine samples.

Variable results were seen in reduction of TV based on leaf age and axis with the high concentration treatment of SH (**Figure 3D**). In 15-day fresh and replicast romaine, 3.18-4.44 and >2.29 log reductions were recorded, respectively. In 45-day fresh and replicast romaine, >3.71 and 1.31-1.46 log reductions were seen, respectively. Infectious virus was reduced by 200 ppm SH treatment below the limit of detection (0.5 log_10_ PFU) for adaxial 45-day old fresh samples and abaxial 15-day old replicast samples. The trend observed in the high concentration PAA treatment was observed in both SH treatments, with greater viral recovery from replicast romaine samples than fresh.

### 3.5 Silwet L-77 addition on viral inactivation by sanitizers

**Figure 4:**
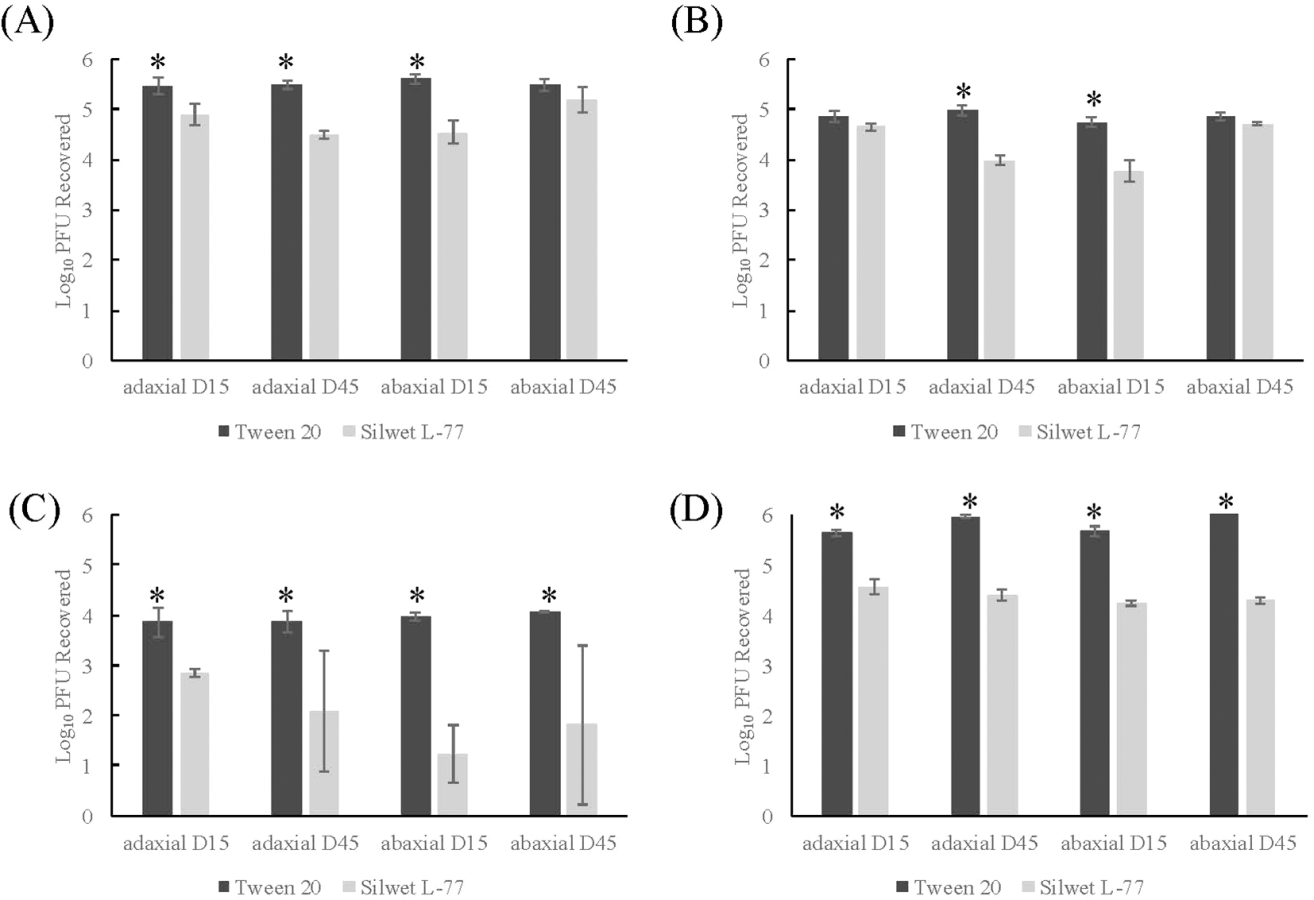
Comparison of TV recovery from Tween 20- and Silwet L-77- supplemented romaine replicasts after two-step washing procedure. **(A)** Control- one-step washing procedure, **(B)** Control- two-step washing procedure, **(C)** Treatment with 50 ppm sodium hypochlorite, **(D)** Treatment with 80 ppm PAA. Log_10_ PFU recovered is log-transformed Tulane virus titer recovered from coupons after a two-step washing procedure. Analysis of variance (ANOVA) with Tukey HSD method *post-hoc* applied to data to determine significance. *Denotes a significantly higher viral recovery (*p*-value <0.05) comparing Tween 20- and Silwet- supplemented replicasts.

To determine if the addition of Silwet L-77 influenced sanitizer efficacy on viral inactivation, 0.2% Silwet L-77 was added to the TV inoculum of romaine replicasts before the two-step washing procedure described above. No significant difference was found between fresh and replicast in either 45-day adaxial or abaxial romaine replicasts. However, significant differences were found between 15-day fresh and replicast adaxial and abaxial surfaces. Viral recovery from Silwet L-77- supplemented replicasts was significantly impacted by both leaf age and leaf axis. Viral recovery from romaine replicasts after all sanitizer treatments was found to be significantly lower with Silwet L-77 addition than Tween 20 addition (*p <*0.0001) (**Figure 4**). It was found that Silwet L-77 addition led to decreased viral recovery for every leaf age and axis combination tested, including both 15-day adaxial and abaxial, as well as 45-day adaxial and abaxial replicasts (*p <*0.0001). In Silwet L-77- supplemented replicasts, treatment with 50 ppm SH led to a 1.81-2.9 log reduction in TV recovery compared to the control (**Figure 4C**), and treatment with 200 ppm led to no recovered virus from replicasts. Treatment with 20 and 80 ppm PAA did not significantly impact recovered virus compared to the control (**Figure 4D**).

## 4 Discussion

In both fresh and replicast spinach and romaine lettuce, viral recovery was significantly enhanced using the mock sanitizer process (two-step wash) compared to a single washing step (*p*-value <0.0001). It was found that a one-step washing procedure led to significant differences in viral recovery between fresh and replicast samples. Adopting a two-step washing procedure by adding a mock sanitization step impacted viral recovery from samples by reducing dissimilarities between fresh and replicast samples and allowing for comparable viral recovery between fresh samples and their respective replicasts.

An inherent quality of polydimethylsiloxane is a hydrophobic surface, meaning it has a contact angle of greater than 90° and up to 180°. Fresh leafy greens typically have a hydrophilic surface with a contact angle of less than 90°. A lower contact angle leads to improved surface wettability, allowing for greater contact between the inoculum and the plant surface. To determine if and what surface modifications PDMS replicasts would require in order to have comparable wettability to fresh plant tissue, the contact angles of fresh and replicast leaves were measured and compared. It was found that 0.05% Tween 20 addition to viral inoculum of romaine replicasts was able to decrease the contact angle to the range found in fresh romaine samples. The addition of a “super spreader” surfactant (Silwet L-77) (Gu et al., 2011) to viral inoculum of PDMS replicasts effectively reduced the contact angle of PBS droplets on flat replicasts (no surface topography) from 107.5° to 24.4°, a significantly larger reduction in contact angle than the addition of Tween 20 to droplets on flat replicasts which measured a contact angle of 74.4°. A 50° difference in contact angle was measured between the two surfactants, meaning that addition of Silwet L-77 leads to the greatest wettability of PDMS surfaces. As organosilicone surfactants like Silwet L-77 are commonly added to agro-chemical products, this could be a useful tool in modeling the influence of herbicide or pesticide addition to the phyllotelma on foodborne virus removal with a chemical sanitizer washing procedure. The addition of these agro-chemical products may further impact the efficacy of chemical sanitizers on viral inactivation, as shown with this preliminary data on romaine replicasts. We found that sanitizer efficacy was improved with the addition of Silwet L-77 to the romaine inoculum with less recovery of infectious virus from samples, likely due to increased contact between waterscape and leaf surface as a result of improved surface wettability, or inherent virucidal qualities of Silwet L-77. This suggests that surfactant addition could further improve inactivation of foodborne viruses when combined with chemical sanitizers, aligning with previous work (Predmore and Li, 2011) and a notable area in need of further research.

In general, inactivation of Tulane virus by chemical sanitizers was not impacted by leaf axis regardless of leafy green (spinach, romaine) or leaf material (PDMS replicast or fresh) sampled. For romaine samples, it was found that there was no significant difference in log_10_ PFU recovered based on leaf age or axis across fresh and replicast samples. For spinach, it was found that there was no significant difference in log_10_ PFU recovered based on leaf axis across fresh and replicast samples, but there was a significant difference based on leaf age (*p*-value < 2.2 e-16). In romaine samples, topographic complexity did not influence sanitizer virucidal effects. Within replicast samples, TV recovery in the control and high concentrations of sanitizer were significantly impacted by leaf age, with greater recovery recorded from older leaves. Viral recovery from all fresh samples were significantly impacted by leaf age with greater recovery recorded from younger leaves.

In contrast, it was found that leaf age had an impact on sanitizer efficacy in spinach samples. Older leaves, 45 days post-germination, had greater recovery of residual infectious virus after the two-step washing procedure in both fresh and replicast spinach samples. In spinach replicast samples, TV recovery in the high concentrations of both sanitizers and the low concentration of peracetic acid was significantly impacted by leaf age, with greater recovery recorded from older leaves. Viral recovery from all fresh samples were significantly impacted by leaf age with greater recovery recorded from older leaves. The greater degree of surface complexity associated with older leaves has previously been shown to improve pathogen persistence (Esseili et al., 2012; Kroupitski et al., 2011), and both macro- and micro-scale surface structures have been shown to act as harborage sites for pathogens (Yi et al., 2022). Our findings align with this published work for spinach samples, but age of romaine samples did not have a significant impact on virus recovery.

In the context of leafy greens, the leaf surface waterscape has been referred to as the phyllotelma (Doan and Leveau, 2015). The term phyllotelma has been adopted to include the waterscape of all above ground portions of a plant. The shape and volume of the phyllotelma on produce is dynamic. Multiple processes such as precipitation, overhead irrigation, foliar sprays, and evaporation have an impact. The topography of produce surfaces has a significant impact on the size and shape of the phyllotelma (Doan et al., 2020b). Previous work on bacterial interactions with the phyllotelma has shown that *Pseudomonas agglomerans* is most resistant to removal from spinach, and *Escherichia coli* is more resistant to removal at 24°C than 4°C, from older leaves compared to younger leaves, and from abaxial leaf surfaces compared to adaxial leaf surfaces (Doan et al., 2020a). Notably, they did not find any influence of produce surface topography or temperature on the removal of *Bacillus velezensis* spores or *Saccharomyces cerevisiae* yeast particles. As foodborne viruses are more comparable in size to spores and yeast particles than bacterial cells and also lack the motility of bacteria, their published results are consistent with our findings that surface topography has limited impact on virus removal from leafy greens. Their finding that variation in leaf axis does not influence nanoscale (below 100 nanometers) microbial recovery aligns with our findings in relation to foodborne virus recovery.

Previous studies have been conducted to characterize the inactivation of foodborne viruses using SH and PAA, two chemical sanitizers commonly used in the produce industry pre- and post-harvest. These sanitizers are typically added to submersion tanks in processing facilities where leafy greens are washed after harvest. Although the maximum concentration of chlorine that can be added to these tanks is 200 ppm, organic material present in the wash water is able to react with chlorine to reduce the sanitizer’s efficacy by decreasing the amount of active or “free” chlorine available (Fuzawa et al., 2019). This means wash water has to be continuously monitored to ensure proper sanitizer concentration, a costly and inefficient process. Though the efficacy of PAA is less influenced by organic material present, it has also been shown to have limited efficacy on human norovirus surrogates and other foodborne viruses (Fuzawa et al., 2020; Hirneisen and Kniel, 2013). These chemical sanitizers have been shown to be effective at reducing or eliminating bacterial pathogens at these recommended concentrations, however, they are notably less effective at reducing infectious virus quantities (Cromeans et al., 2014; Chiu et al., 2015). All chemical sanitizers employed in this study resulted in recovery of infectious virus after treatment. Both low and high concentrations of SH treatment resulted in some degree of reduction of infectious virus while neither PAA treatment led to any reduction in virus recovery. This could be due to the difference in disinfection mechanisms utilized by these two sanitizers. While both sanitizers damage viral capsid proteins, thereby impacting host receptor binding, peracetic acid has been shown to cause aggregation of TV particles due to its low pH, improving viral resistance to disinfection (Fuzawa et al., 2020). This could explain why both fresh and replicast samples experience little reduction in infectious virus with PAA treatment. It also supports that commonly available commercial sanitizers are ineffective at completely inactivating viral pathogens of concern in the produce industry, aligning with data previously published on the limited impact of chemical sanitizers on human norovirus surrogates.

In summary, mature spinach leaves prove to be the best model system for PDMS casting due to high fidelity between fresh leaf tissue and replicasts. While physical produce characteristics may have some impact on human norovirus surrogate inactivation and removal, it is likely that other factors, particularly specific and non-specific binding mechanisms, are also at play. However, PDMS replicasts, particularly with hydrophobicity modifications, serve as a useful tool to further elucidate the role of produce surface topography on foodborne virus adhesion to and removal from leafy green surfaces.

## Data Availability

The raw data supporting the conclusions of this article will be made available by the authors, without undue reservation.
